# Supplementary material for: The implementation of sex-and gender-based considerations in exercise-based randomized controlled trials in individuals with stroke: A cross-sectional study
Source: PLoS One. 2024 Oct 9;19(10):e0308519. doi: 10.1371/journal.pone.0308519 (PMC11463778; doi:10.1371/journal.pone.0308519)
Supplement: S1 Table — (DOCX) [file pone.0308519.s001.docx]

| Exercise-based randomized controlled trials published prior to and including December 31, 2016 (PRE-SAGER) adhering to each section of the SAGER guidelines. | | | | | | | | | | |
| --- | --- | --- | --- | --- | --- | --- | --- | --- | --- | --- |
| **Article** | **Country** | **Baseline**  **N** | **Proportion of Females** | **Intervention type** | **Title**  **/Abstract** | **Intro** | **Methods** | **Results** | **Discussion** | **Terminology** |
| Ada 2003 | Australia | 27 | 8 (29.6%) | Aerobic | NA | N | N | N | N | N |
| Ada 2013 | Australia | 102 | 31 (30.4%) | Aerobic | NA | N | N | N | N | N |
| Aidar 2012 | Brazil | 24 | 9 (37.5%) | Strength | NA | N | N | N | N | Y |
| Aidar 2014 | Brazil | 24 | 9 (37.5%) | Strength | NA | N | N | N | N | N |
| Aidar 2016 | Brazil | 24 | 9 (37.5%) | Strength | NA | N | N | N | N | N |
| Akbari 2006 | Iran | 34 | 15 (44.1%) | Mixed | NA | N | N | N | N | Y |
| Bale 2008 | Norway | 18 | 11 (61.1%) | Functional | NA | N | N | N | N | N |
| Bateman 2001 | UK | 157 | 60 (38.2%) | Aerobic | NA | N | N | N | N | N |
| Batchelor 2012 | New Zealand | 156 | 57 (36.5%) | Functional | NA | N | N | N | N | Y |
| Bhatia 2014 | India | 30 | NR | Strength | NA | N | N | N | N | N |
| Boyne 2016 | USA | 16 | 7 (43.8%) | Aerobic | NA | N | N | N | N | Y |
| Büyükvural 2015 | Turkey | 50 | 17 (34.0%) | Strength | NA | N | N | N | N | N |
| Britton 2008 | UK | 18 | 4 (22.2%) | Strength | NA | N | N | N | N | N |
| Carda 2013 | Italy | 38 | NR | Aerobic | NA | N | N | N | N | NR |
| Carr 2003 | USA | 40 | 18 (45.0%) | Mixed | NA | N | N | N | N | N |
| Chan 2012 | Australia | 14 | 2 (14.3%) | Mixed | NA | N | N | N | N | N |
| Chen 2015 | Taiwan | 24 | 11 (45.8%) | Strength | NA | N | N | N | N | N |
| Chu 2004 | Canada | 12 | 1 (8.3%) | Aerobic | NA | N | N | N | N | N |
| Clark 2013 | USA | 34 | 9 (26.5%) | Strength | NA | N | N | N | N | Y |
| Combs-Miller 2014 | USA | 20 | 9 (45.0%) | Aerobic | NA | N | N | N | N | N |
| Cooke 2010 | UK | 109 | 44 (40.4%) | Mixed | NA | N | N | N | N | N |
| Corti 2012 | USA | 14 | 2 (14.3 %) | Group 1: Functional  Group 2: Strength | NA | N | N | N | N | N |
| Teixeira da Cunha 2001 | USA | 12 | 0 (0%) | Aerobic | N | N | N | N | N | N |
| Teixaira da Cunha 2002 | USA | 13 | 0 (0%) | Aerobic | N | N | N | N | N | N |
| da Silva  2015 | Brazil | 20 | 13 (65.0%) | Functional | NA | N | N | N | N | N |
| Dean 2000 | Canada | 12 | 5 (41.7%) | Functional | NA | N | N | N | N | N |
| Dean 2012 | Australia | 151 | 73 (48.3%) | Functional | NA | N | N | N | N | N |
| Dean 2014 | Australia | 68 | 25 (36.8%) | Aerobic | NA | N | N | N | N | N |
| Donaldson 2009 | UK | 30 | 17 (56.7%) | Functional | NA | N | N | N | N | N |
| Duncan 1998 | USA | 20 | NR | Mixed | NA | N | N | N | N | NR |
| Duncan 2003 | USA | 92 | 42 (45.7%) | Mixed | NA | N | N | N | N | Y |
| Eich 2004 | Germany | 50 | 17 (34.0%) | Aerobic | NA | N | N | N | N | Y |
| Ellis 2009 | USA | 14 | 5 (35.7%) | Functional | NA | N | N | N | N | N |
| Engardt 1995 | Sweden | 20 | 5 (25.0%) | Strength | NA | N | N | N | N | N |
| English 2015 | Australia | 283 | 116 (41.0%) | Functional | NA | N | N | N | N | N |
| Faulkner 2013 | New Zealand | 60 | 29 (48.3%) | Mixed | NA | N | N | N | N | N |
| Faulkner 2015 | New Zealand | 55 | 26 (47.3%) | Mixed | NA | N | N | N | N | N |
| Fernandes 2015 | Portugal | 16 | 0 (0%) | Functional | N | N | N | N | N | N |
| Fernandez-Gonzalo 2016 | Spain | 29 | 7 (24.0%) | Strength | NA | N | N | N | N | Y |
| Flansbjer 2008 | Sweden | 24 | 10 (41.7%) | Strength | NA | N | Y | N | N | N |
| Furnari 2014 | Italy | 40 | 20 (40%) | Functional | NA | N | Y | N | N | N |
| Galvin 2011 | Ireland | 40 | 20 (50.0%) | Functional | NA | N | N | N | N | Y |
| Glasser 1986 | USA | 20 | 10 (50.0%) | Strength | NA | N | N | N | N | Y |
| Globas 2012 | Germany | 36 | 7 (19.4%) | Aerobic | NA | N | N | N | N | N |
| Gordon 2013 | Jamaica | 128 | 70 (54.7%) | Aerobic | NA | N | N | N | N | Y |
| Graef 2016 | Brazil | 27 | 16 (59.3%) | Group 1: Functional  Group 2: Strength | NA | N | N | N | N | N |
| Harrington 2010 | UK | 243 | 111 (45.7%) | Functional | NA | N | N | N | N | Y |
| Holmgren 2010a | Sweden | 34 | 13 (38.2%) | Functional | NA | N | N | N | N | N |
| Holmgren 2010b | Sweden | 34 | 13 (38.2%) | Functional | NA | N | N | N | N | N |
| Hornby 2015 | USA | 32 | 8 (25.0%) | Aerobic | NA | N | N | N | N | N |
| Inaba 1973 | USA | 77 | 40 (51.9%) | Mixed | NA | N | N | N | N | Y |
| Ivey 2007 | USA | 69 | NR | Aerobic | NA | N | N | N | N | N |
| Ivey 2010 | USA | 53 | 24 (45.3%) | Aerobic | NA | N | N | N | N | N |
| Ivey 2015 | USA | 34 | 13 (38.2%) | Aerobic | NA | N | N | N | N | N |
| Jin 2012 | China | 133 | 39 (29.3%) | Aerobic | NA | N | N | N | N | N |
| Jin 2013 | China | 128 | 37 (28.9%) | Aerobic | NA | N | Y | N | N | N |
| Katz-Laurer 2003a | Israel | 92 | 42 (45.7%) | Aerobic | NA | N | N | N | N | N |
| Katz-Laurer 2003b | Israel | 92 | 42 (45.7%) | Aerobic | NA | N | Y | Y | N | N |
| Katz-Laurer 2006 | Israel | 24 | 11 (45.8%) | Aerobic | NA | N | N | N | N | N |
| Katz-Laurer 2007 | Israel | 64 | 31 (48.4%) | Aerobic | NA | N | N | N | N | N |
| Keerthi Chandra Sekhar 2013 | India | 40 | NR | Strength | NA | N | N | N | N | N |
| Kim 2001 | Canada | 20 | 6 (30.0%) | Strength | NA | N | Y | N | N | N |
| Kim 2014a | South Korea | 28 | 0 (0%) | Functional | N | N | N | N | N | N |
| Kim 2014b | South Korea | 20 | NR | Mixed | NA | N | N | N | N | NA |
| Kim 2015 | South Korea | 32 | 7 (21.9%) | Aerobic | NA | N | N | N | N | N |
| Kim 2016 | South Korea | 20 | 7 (35.0%) | Mixed | NA | N | N | N | N | N |
| Koc 2015 | Turkey | 72 | NR | Functional | NA | N | N | N | N | N |
| Kuys 2011 | Australia | 30 | 18 (60.0%) | Aerobic | NA | N | N | N | N | N |
| Lai 2006 | USA | 93 | 43 (46.2%) | Mixed | NA | N | N | N | N | Y |
| Langhammer 2007 | Norway | 75 | NR | Mixed | NA | N | Y | N | N | N |
| Langhammer 2008 | Norway | 75 | 32 (42.7%) | Mixed | NA | N | Y | N | N | N |
| Langhammer 2009 | Norway | 75 | 32 (42.7%) | Mixed | NA | N | Y | N | N | N |
| Langhammer 2010 | Norway | 39 | 23 (59.0%) | Mixed | NA | N | N | N | N | N |
| Lau 2011 | China | 30 | 9 (30.0%) | Aerobic | NA | N | N | N | N | N |
| Leddy 2016 | USA | 33 | 10 (30.3%) | Aerobic | NA | N | N | N | N | N |
| Lee 2008 | Australia | 48 | 20 (41.7%) | Group 1: Aerobic  Group 2:  Strength  Group 3:  Mixed (Strength + Aerobic) | NA | N | N | N | N | N |
| Lee 2010 | Australia | 48 | 20 (41.7%) | Group 1: Aerobic  Group 2:  Strength  Group 3:  Mixed  (Strength + Aerobic) | NA | N | N | N | N | N |
| Lee 2013a | South Korea | 33 | 13 (39.4%) | Strength | NA | N | N | N | N | Y |
| Lee 2013b | South Korea | 39 | 14 (35.9%) | Strength | NA | N | N | N | N | Y |
| Lee 2015a | South Korea | 26 | NR | Mixed | NA | N | Y | N | N | NR |
| Lee 2015b | South Korea | 61 | 25 (41.0%) | Aerobic | NA | N | N | N | N | N |
| Lee 2013c | South Korea | 28 | 11 (39.3%) | Strength | NA | N | N | N | N | Y |
| Lennon 2008 | Ireland | 48 | 20 (41.7%) | Aerobic | NA | N | Y | Y | N | Y |
| Letombe 2010 | France | 18 | 7 (38.9%) | Mixed | NA | N | N | N | N | N |
| Lui-Ambrose 2015 | Canada | 25 | 10 (40.0%) | Functional | NA | N | N | N | Y | Y |
| Luft 2008 | USA | 71 | 38 (53.5%) | Aerobic | NA | N | Y | Y | N | N |
| Mackay-Lyons 2013 | Canada | 50 | 21 (42.0%) | Aerobic | NA | N | N | N | N | N |
| Macko 2005 | USA | 61 | 18 (29.5%) | Aerobic | NA | N | N | N | N | N |
| Malagoni 2016 | Italy | 12 | 3 (25.0%) | Aerobic | NA | N | N | N | N | N |
| Mares 2014 | UK | 52 | 18 (42.9%) | Functional | NA | N | N | N | N | N |
| Marsden 2016 | Australia | 20 | 12 (60.0%) | Aerobic | NA | N | N | N | N | N |
| Marigold 2005 | Canada | 48 | 13 (27.0%) | Functional | NA | N | N | N | N | N |
| Mayo 2013 | Canada | 87 | 27 (31.0%) | Aerobic | NA | N | Y | Y | Y | N |
| Mead 2007 | UK | 66 | 30 (45.5%) | Mixed | NA | N | Y | N | N | N |
| Milot 2013 | Canada | 30 | 12 (40%) | Strength | NA | N | N | N | N | N |
| Monticone 2013 | Italy | 60 | 26 (43.3%) | Functional | NA | N | N | N | N | Y |
| Moore 2010 | USA | 20 | 6 (30.0%) | Aerobic | NA | N | N | N | N | N |
| Moore 2015 | UK | 40 | 6 (15.0%) | Functional | NA | N | N | N | Y | N |
| Moore 2016 | UK | 40 | 5 (15.0%) | Functional | NA | N | N | N | Y | N |
| Moreland 2003 | Canada | 133 | 52 (39.1%) | Strength | NA | N | N | N | N | Y |
| Mudge 2009 | New Zealand | 58 | 26 (44.8%) | Functional | NA | N | N | N | N | Y |
| Nadeau 2013 | USA | 408 | 184 (45.1%) | Mixed | NA | N | N | N | N | N |
| Nilsson 2001 | Sweden | 73 | 33 (45.2%) | Aerobic | NA | N | N | N | N | Y |
| Olawale 2011 | Ghana | 60 | 26 (43.3%) | Aerobic | NA | N | N | N | N | Y |
| Olney 2006 | Canada | 72 | 27 (37.5%) | Strength | NA | N | Y | Y | Y | N |
| Ouellette 2004 | USA | 42 | 14 (33.3%) | Strength | NA | N | N | N | N | N |
| Outermans 2010 | Netherlands | 43 | 7 (16.3%) | Functional | NA | N | N | N | N | N |
| Page 2008 | USA | 7 | 2 (28.6%) | Aerobic with resistive component (Used Nustep machine) | NA | N | N | N | N | Y |
| Pang 2005 | Canada | 63 | 26 (41.3%) | Functional | NA | N | Y | N | N | N |
| Pang 2006a | Canada | 63 | 21 (33.3%) | Functional | NA | N | Y | Y | Y | N |
| Pang 2006b | Canada | 63 | 26 (41.3%) | Functional | NA | N | Y | N | N | N |
| Park 2011 | South Korea | 25 | 13 (52.0%) | Aerobic | NA | N | N | N | N | N |
| Patten 2013 | USA | 19 | 4 (21.1%) | Strength | NA | N | N | N | N | N |
| Pohl 2002 | Germany | 60 | 17 (28.3%) | Aerobic | NA | N | N | N | N | Y |
| Potempa 1995 | USA | 42 | 19 (45.2%) | Aerobic | NA | N | N | N | N | N |
| Quaney 2009 | USA | 38 | 21 (55.2%) | Aerobic | NA | N | N | N | N | N |
| Richards 1993 | Canada | 27 | NR | Mixed | NA | N | N | N | N | N |
| Richards 2004 | Canada | 63 | 20 (31.7%) | Mixed | NA | N | N | N | N | N |
| Rimmer 2009 | USA | 55 | 33 (60.0%) | Aerobic | NA | N | N | N | N | N |
| Salbach 2004 | Canada | 91 | 35 (38.5%) | Functional | NA | N | N | N | N | N |
| Salbach 2005 | Canada | 83 | 31 (37.3%) | Functional | NA | N | Y | Y | N | Y |
| Sandberg 2016 | Sweden | 56 | 28 (50.0%) | Aerobic | NA | N | N | N | N | N |
| Severinsen 2014 | Denmark | 43 | 12 (27.9%) | Group 1: Aerobic  Group 2: Strength | NA | N | N | N | N | Y |
| Shaughnessy 2012 | USA | 64 | 36 (56.3%) | Aerobic | NA | N | N | N | N | N |
| Shin 2011 | South Korea | 21 | 13 (61.9%) | Mixed | NA | N | N | N | N | Y |
| Sims 2009 | Australia | 45 | 18 (40.0%) | Strength | NA | N | N | N | N | N |
| Smith 2008 | USA | 20 | 8 (40.0%) | Aerobic | NA | N | N | N | N | N |
| Son 2014 | South Korea | 28 | 13 (46.4%) | Strength | NA | N | N | N | N | Y |
| Studenski 2005 | USA | 93 | 43 (46.2%) | Functional | NA | N | N | N | N | N |
| Sullivan 2007 | USA | 80 | 35 (43.8%) | Strength | NA | N | N | N | N | N |
| Takami 2010 | Japan | 36 | 16 (44.4%) | Aerobic | NA | N | N | N | N | N |
| Tang 2014 | Canada | 50 | 21 (42.0%) | Aerobic | NA | N | Y | N | N | N |
| Tang 2016 | Canada | 50 | 21 (42.0%) | Aerobic | NA | N | N | N | N | N |
| Teixeira-Salmela 1999 | Canada | 13 | 6 (46.2%) | Mixed | NA | N | N | N | N | N |
| Thielman 2004 | USA | 12 | 7 (58.3%) | Strength | NA | N | N | N | N | Y |
| Toledano-Zarhi 2011 | Israel | 28 | 7 (25.0%) | Aerobic | NA | N | N | N | N | N |
| Topcuoglu 2015 | Turkey | 40 | 18 (45.0%) | Aerobic | NA | N | N | N | N | N |
| Treger 2014 | Israel | 56 | 18 (32.1%) | Functional | NA | N | N | N | N | N |
| Tung 2010 | Taiwan | 32 | 12 (37.5%) | Functional | NA | N | N | N | N | N |
| van de Port 2012 | Netherlands | 250 | 88 (35.2%) | Functional | NA | N | N | N | N | N |
| Wang 2014a | China | 48 | 13 (27.1%) | Aerobic | NA | N | N | N | Y | N |
| Wang 2014b | China | 54 | 18 (33.3%) | Aerobic | NA | N | Y | Y | N | Y |
| Winstein 2004 | USA | 60 | 27 (45.0%) | Group 1: Functional  Group 2: Strength | NA | N | N | N | N | N |
| Yang 2006 | Taiwan | 48 | 16 (33.3%) | Strength | NA | N | N | N | N | N |
| Yang 2014 | Taiwan | 30 | 8 (26.7%) | Aerobic | NA | N | Y | N | N | Y |
| Yoo 2011 | South Korea | 28 | 11 (39.3%) | Functional | NA | N | N | N | N | N |
| Zedlitz 2012 | Netherlands | 83 | 40 (48.2%) | Mixed | NA | N | N | N | N | N |
| Zhang 2016 | China | 36 | 19 (52.8%) | Functional | NA | N | N | N | N | Y |
| Zhu 2016 | China | 28 | 6 (21.4%) | Functional | NA | N | N | N | N | N |
| Zou 2015 | China | 56 | 34 (60.7%) | Strength | NA | N | N | N | N | Y |

*Abbreviations.* N=No; Y=Yes; NA= Not Applicable; NR= Not Reported; USA= United States of America; UK= United Kingdom

| Exercise-based randomized controlled trials published between 2017- March 2023 (POST-SAGER) adhering to each section of the SAGER guidelines. | | | | | | | | | | |
| --- | --- | --- | --- | --- | --- | --- | --- | --- | --- | --- |
| **Article** | **Country** | **Baseline**  **N** | **Proportion of Females** | **Intervention type** | **Title**  **/Abstract** | **Intro** | **Methods** | **Results** | **Discussion** | **Terminology** |
| Antonio 2023 | Brazil | 26 | 13 (50%) | Mixed | NA | N | N | N | N | N |
| Aguiar 2020 | Brazil | 22 | 6 (9.1%) | Aerobic | NA | N | N | N | N | N |
| Aidar 2018 | Brazil | 36* | 17(47.2%)* | Aerobic | NA | N | N | N | Y | Y |
| Alipsatici 2020 | Turkey | 28 | 11 (39.3%) | Aerobic | NA | N | N | N | N | Y |
| Agni 2017 | Mumbai | 37 | 8 (21.6%) | Strength | NA | N | N | N | N | N |
| Arabzadeh 2018 | Iran | 20 | 5 (25.0%) | Functional | NA | N | N | N | N | N |
| Aravind 2022 | Canada | 33 | 15 (45.5%) | Functional | NA | N | N | N | N | N |
| Aung 2022 | Myanmar | 13 | 14 (35%) | Functional | NA | N | N | N | N | Y |
| Bo 2019 | China | 178 | 79 (44.4%) | Mixed | NA | N | N | N | N | Y |
| Brauer 2022 | Australia | 119 | 25 (21.0%) | Aerobic | NA | N | N | N | N | Y |
| Brito 2020 | Brazil | 22 | NR | Aerobic | NA | N | N | N | N | NR |
| Boyne 2023 | USA | 55 | 19 (34.5%) | Aerobic | NA | N | N | N | N | Y |
| Chan 2017 | Canada | 25 | 12 (48.0%) | Mixed | NA | N | Y | N | Y | N |
| Chang 2021 | Taiwan | 16 | 5 (31.3%) | Aerobic | NA | N | N | N | N | N |
| Cheng 2020 | Taiwan | 18 | 4 (22.2%) | Aerobic | NA | N | Y | N | N | Y |
| Coroian 2018 | France | 20 | 4 (20.0%) | Strength | NA | N | N | N | N | Y |
| da Rosa Pinheira 2021 | Brazil | 20 | 12 (60.0%) | Aerobic | NA | N | N | N | N | N |
| Dean 2018 | UK | 45 | 15 (33.3%) | Functional | NA | N | N | N | N | N |
| Debrecini-Nagy 2019 | Hungary | 35 | 11 (31.4%) | Aerobic | NA | N | N | N | N | N |
| Dehno 2021 | Iran | 26 | 13 (50%) | Strength | NA | N | N | N | N | Y |
| Deijle 2022 | Netherlands | 119 | 49 (41.2%) | Mixed | NA | N | Y | N | N | Y |
| da Sousa 2019 | Australia | 30 | 13 (43.3%) | Functional | NA | N | N | N | N | N |
| English 2018 | Australia | 19 | 9 (47.4%) | Aerobic | NA | N | Y | N | N | Y |
| Eyvaz 2018 | Turkey | 60 | 29 (48.3%) | Mixed | NA | N | N | N | N | N |
| Faulkner 2017 | New Zealand | 47 | 12 (25.5%) | Aerobic | NA | N | N | N | N | N |
| Fonseca 2023 | Brazil | 7 | NR | Mixed | NA | N | N | N | Y | Y |
| Franciulli 2019 | Brazil | 12 | 10 (83.3%) | Aerobic | NA | N | N | N | N | Y |
| Gambassi 2019 | Brazil | 22 | 13 (59.1%) | Strength | NA | N | N | N | N | N |
| Gjellesvik 2020 | Norway | 70 | 29 (41.4%) | Aerobic | NA | N | N | N | N | N |
| Gjellesvik 2021 | Norway | 70 | 29 (41.4%) | Aerobic | NA | N | N | N | Y | N |
| Gu 2022 | China | 56 | 26 (46.4%) | Strength | NA | N | N | N | N | N |
| Han 2018 | South Korea | 20 | 8 (40.0%) | Aerobic | NA | N | N | N | N | N |
| Hendrey 2018 | Australia | 30 | 14 (46.7%) | Strength | NA | N | N | N | N | Y |
| Hogg 2020 | Germany | 43 | 18 (41.2%) | Strength | NA | N | N | N | N | Y |
| Hornby 2019 | USA | 90 | 32 (35.6%) | Aerobic | NA | N | N | N | N | Y |
| Horvath 2022 | Hungary | 37 | NR | Aerobic | NA | N | N | N | N | Y^a^ |
| Hsu 2019 | Taiwan | 30 | 5 (16.7%) | Aerobic | NA | N | N | N | N | N |
| Hsu 2021 | Taiwan | 23 | 3 (13. 0%) | Aerobic | NA | N | N | N | N | N |
| Iqbal 2020 | Pakistan | 64 | 30 (46.9%) | Functional | NA | N | N | N | N | N |
| Ivey 2017 | USA | 30 | 9 (30.0%) | Strength | NA | N | N | N | N | N |
| Jeon 2018 | South Korea | 20 | 7 (35.0%) | Strength | NA | N | N | N | N | N |
| Kerr 2017 | UK | 93 | 39 (41.9%) | Functional | NA | N | N | N | N | Y |
| Khattab 2020 | Canada | 50 | 21 (42.0%) | Aerobic | NA | Y | Y | Y | Y | Y |
| Khan 2021 | Pakistan | 48 | 22 (46%) | Functional | NA | N | N | N | N | Y |
| Kim 2017 | South Korea | 29 | 10 (34.5%) | Mixed | NA | N | N | N | N | N |
| Kim 2022 | South Korea | 22 | 6 (27.3%) | Aerobic | NA | N | N | N | N | Y |
| Klassen 2020 | Canada | 74 | 30 (40.5%) | Aerobic | NA | N | Y | N | N | Y |
| Knox 2018 | South Africa | 144 | 72 (50.0%) | Group 1: Strength  Group 2: Functional | NA | N | N | N | N | N |
| Koch 2020 | USA | 131 | 50 (38.0%) | Mixed | NA | N | Y | N | N | N |
| Krawcyk 2019 | Denmark | 63 | 14 (22.0%) | Aerobic | N | N | N | N | Y | N |
| Lamberti 2017 | Italy | 35 | 8 (22.9%) | Mixed | NA | N | N | N | N | Y |
| Lapointe 2023 | Canada | 52 | 19 (36.5%) | Aerobic | NA | N | Y | N | N | N |
| Lee 2017 | South Korea | 20 | 5 (25.0%) | Strength | NA | N | N | N | N | N |
| Lee 2018 | South Korea | 37 | 18 (48.6%) | Aerobic | NA | N | N | N | N | N |
| Lee 2019 | South Korea | 45 | 12 (26.7%) | Aerobic | NA | N | N | N | N | N |
| Linder 2017 | USA | 17 | 2 (11.8%) | Aerobic | NA | N | N | N | N | N |
| Linder 2019 | USA | 40 | 11 (27.8%) | Aerobic | NA | N | N | N | N | Y |
| Linder 2021 | USA | 43 | 10 (23.3%) | Aerobic | NA | N | Y | N | N | N |
| Lui- Ambrose 2022 | Canada | 120 | 46 (38.3%) | Functional | NA | N | N | N | N | Y |
| Lund 2018 | Denmark | 43 | 12 (27.9%) | Group 1: Aerobic  Group 2: Strength | NA | N | N | N | N | Y |
| Marzolini 2018 | Canada | 68 | 24 (35.3%) | Group 1:  Mixed (Strength + Aerobic)  Group 2:  Aerobic | NA | N | Y | Y | N | N |
| MacKay-Lyons 2022 | Canada | 184 | 63 (34.2%) | Mixed | NA | N | N | N | N | N |
| Michalski 2023 | Brazil | 7 | 3 (42.8%) | Mixed | NA | N | N | N | Y | Y |
| Najafabadi 2019 | Iran | 42 | 18 (42.9%) | Functional | NA | N | N | N | N | N |
| Milot 2019 | Canada | 12 | NR | Group 1: Strength  Group 2: Functional | NA | N | N | N | N | NR |
| Munari 2018 | Italy | 15 | 1 (6.7%) | Aerobic | NA | N | N | N | N | N |
| Nave 2019 | Germany | 200 | 81 (41.0%) | Aerobic | NA | N | Y | Y | Y | N |
| Nepveu 2017 | Canada | 22 | 6 (27.3%) | Aerobic | NA | N | N | N | N | Y |
| Ofori 2019 | Ghana | 20 | 8 (40.0%) | Aerobic | NA | N | N | N | N | Y |
| Pallesen 2019 | Denmark | 30 | 16 (53.3%) | Aerobic | NA | N | N | N | N | N |
| Park 2017 | South Korea | 30 | NR | Functional | NA | N | N | N | N | NR |
| Park 2021 | South Korea | 21 | 9 (42.9%) | Functional | NA | N | N | N | N | Y |
| Rackroll 2021 | Germany | 200 | 81 (41.0%) | Aerobic | NA | N | Y | N | N | Y |
| Reynolds 2021 | New Zealand | 20 | 2 (10%) | Aerobic | NA | N | N | N | N | N |
| Ribeiro 2017 | Brazil | 38 | 15 (39.5%) | Aerobic | NA | N | N | N | N | N |
| Rose 2017 | USA | 347 | 158 (45.5%) | Mixed | NA | N | N | N | N | N |
| Rosenfeldt 2019 | USA | 40 | 11 (27.5%) | Mixed | NA | N | Y | N | N | N |
| Saadatnia 2020 | Iran | 40 | 23 (57.8%) | Mixed | NA | N | N | N | N | N |
| Salehi Dehno 2021 | Iran | 26 | 13 (50.0%) | Strength | NA | N | N | N | N | Y |
| Sanchez-Sanchez 2017 | Spain | 15 | 7 (45.6%) | Functional | NA | N | N | N | N | N |
| Sandberg 2020 | Sweden | 52 | 32 (61.5%) | Aerobic | NA | N | N | N | N | Y |
| Sandberg 2021 | Sweden | 52 | 32 (61.5%) | Aerobic | NA | N | N | N | N | N |
| Serra 2019 | USA | 25 | 6 (24.0%) | Aerobic | NA | N | N | N | N | Y |
| Serra 2022 | USA | 39 | 8 (20.5%) | Aerobic | NA | N | N | N | Y | Y |
| Shao 2023 | China | 127 | 43 (33.9%) | Strength | NA | N | N | N | N | N |
| Stuart 2019 | USA | 76 | 37 (48.7%) | Functional | NA | N | N | N | N | N |
| Vahlberg 2017a | Sweden | 67 | 16 (23.9%) | Functional | NA | N | Y | N | N | N |
| Vahlberg 2017b | Sweden | 43 | 10 (23.3%) | Functional | NA | N | N | N | N | N |
| Valkenborghs 2019 | Australia | 20 | 9 (45.0%) | Mixed | NA | N | N | N | N | Y |
| Vanroy 2017 | Belgium | 59 | 21 (35.6%) | Aerobic | NA | N | N | N | N | N |
| Vanroy 2019 | Belgium | 59 | NR | Aerobic | NA | N | N | N | N | NR |
| Wijkman 2018 | Sweden | 53 | 27 (59.0%) | Aerobic | NA | N | Y | Y | N | N |
| Wu 2020 | China | 31 | 9 (29.0%) | Aerobic | NA | N | Y | Y | N | Y |
| Yeh 2019 | Taiwan | 30 | 9 (30.0%) | Aerobic | NA | N | N | N | Y | N |
| Yeh 2022 | Taiwan | 56 | 18 (32.1%) | Aerobic | NA | N | N | N | N | N |

*Note.* *Post intervention (no baseline). *Abbreviations.* N=No; Y=Yes; NA= Not Applicable; NR= Not Reported; USA= United States of America; UK= United Kingdom. ^a^Sex-related terminology used in introduction.
