# Supplementary material for: The implementation of sex-and gender-based considerations in exercise-based randomized controlled trials in individuals with stroke: A cross-sectional study
Source: PLoS One. 2024 Oct 9;19(10):e0308519. doi: 10.1371/journal.pone.0308519 (PMC11463778; doi:10.1371/journal.pone.0308519)
Supplement: Attachment — Submitted filename: Response to reviewers - PLOS One_EW.docx [file pone.0308519.s002.docx]

**Response Document:** “The Implementation of Sex-and Gender-Based Considerations in Exercise-Based Randomized Controlled Trials in Individuals with Stroke: A Cross-Sectional Study”

We thank the reviewers for their time and positive reviews of our study, and for their appreciation of the importance of incorporating sex and gender considerations into research. We have provided a point-by-point response to Reviewer 2’s comments below, and have uploaded a clean and revised manuscript to the portal. Changes to the manuscript have been added in yellow highlight.

**Reviewer 2**

Excellent work!
1. Kindly rephrase line 110 to 114 or simplify the sentence(s) for easy comprehension.

AUTHOR RESPONSE: We thank the reviewer for this suggestion around simplifying the sentence in lines 110-114. The revised wording is provided below.

**Lines 110-114:** However, despite evidence of sex and/or gender differences in the effects of exercise [11-14] and females having poorer outcomes compared to males after stroke [22–25], sex- and gender-based considerations continue to be overlooked in all aspects of research [7,26].

2. Was there any specific reason(s) for the non-inclusion of thesis dissertations?

AUTHOR RESPONSE: We thank the reviewer for this comment. There are a few reasons that thesis dissertations were excluded: 1) We opted to only include studies that were peer reviewed to provide an appraisal of the literature meeting standards of rigour, quality and ethical conduct held by academic journals; 2) The individual studies included in thesis dissertations may have in fact been previously published in peer-reviewed journals, and thus were excluded to avoid the risk of duplication; and 3) Different institutions have varying requirements for the format of thesis dissertations, and thus the appraisal of content within individual dissertations would have been challenging to report.
